# Supplementary material for: A systematic exploration of bacterial form I rubisco maximal carboxylation rates
Source: EMBO J. 2024 May 28;43(14):13. doi: 10.1038/s44318-024-00119-z (PMC11251275; doi:10.1038/s44318-024-00119-z)
Supplement: Supplementary file 1 — Appendix [file 44318_2024_119_MOESM1_ESM.pdf]

Systematic exploration of bacterial form I rubisco maximal carboxylation rates

Benoit de Pins, Lior Greenspoon, Yinon M. Bar-On, Melina Shamshoum, Roee Ben-Nissan, Eliya Milshtein, Dan Davidi, Itai Sharon, Oliver Mueller-Cajar, Elad Noor & Ron Milo

Appendix

Table of Contents

Appendix Figure S1	Page 2
Appendix Figure S2	Page 3
Appendix Figure S3	Page 4
Appendix Figure S4	Page 5
Appendix Figure S5	Page 5
Appendix Figure S6	Page 6
Appendix Figure S7	Page 6
Appendix Figure S8	Page 7
Appendix Figure S9	Page 8
Appendix Figure S10	Page 9
Appendix Figure S11	Page 10
Appendix Figure S12	Page 10
Appendix Figure S13	Page 11
Appendix Table S1	Page 11
Appendix Note S1	Page 12
Appendix Note S2	Page 12
References	Page 15

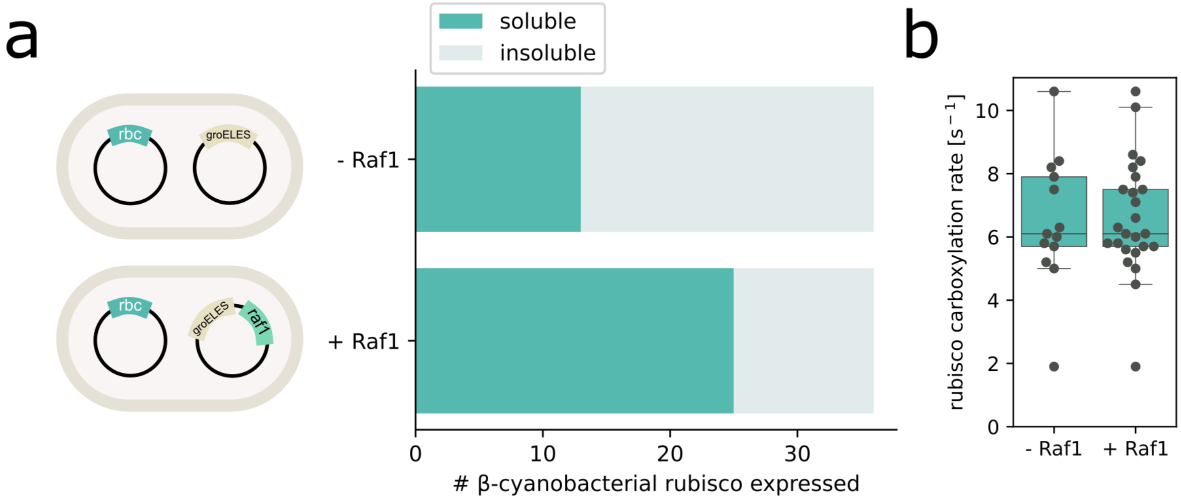

Appendix Figure S1. Co-expression of β-cyanobacteria rubisco with Raf1 from Euhalothece natronophila greatly enhances solubility.

*(**A**) rbcL-rbcX-rbcS operon containing plasmid was cotransformed with a plasmid expressing groEL-groES with or without raf1 in E. coli. The presence of Raf1 almost doubled the number of soluble and active rubiscos among the 36 tested β-cyanobacteria variants. (**B**) The average rate of β-cyanobacterial rubiscos is not changed by adding the newly soluble rubiscos.*

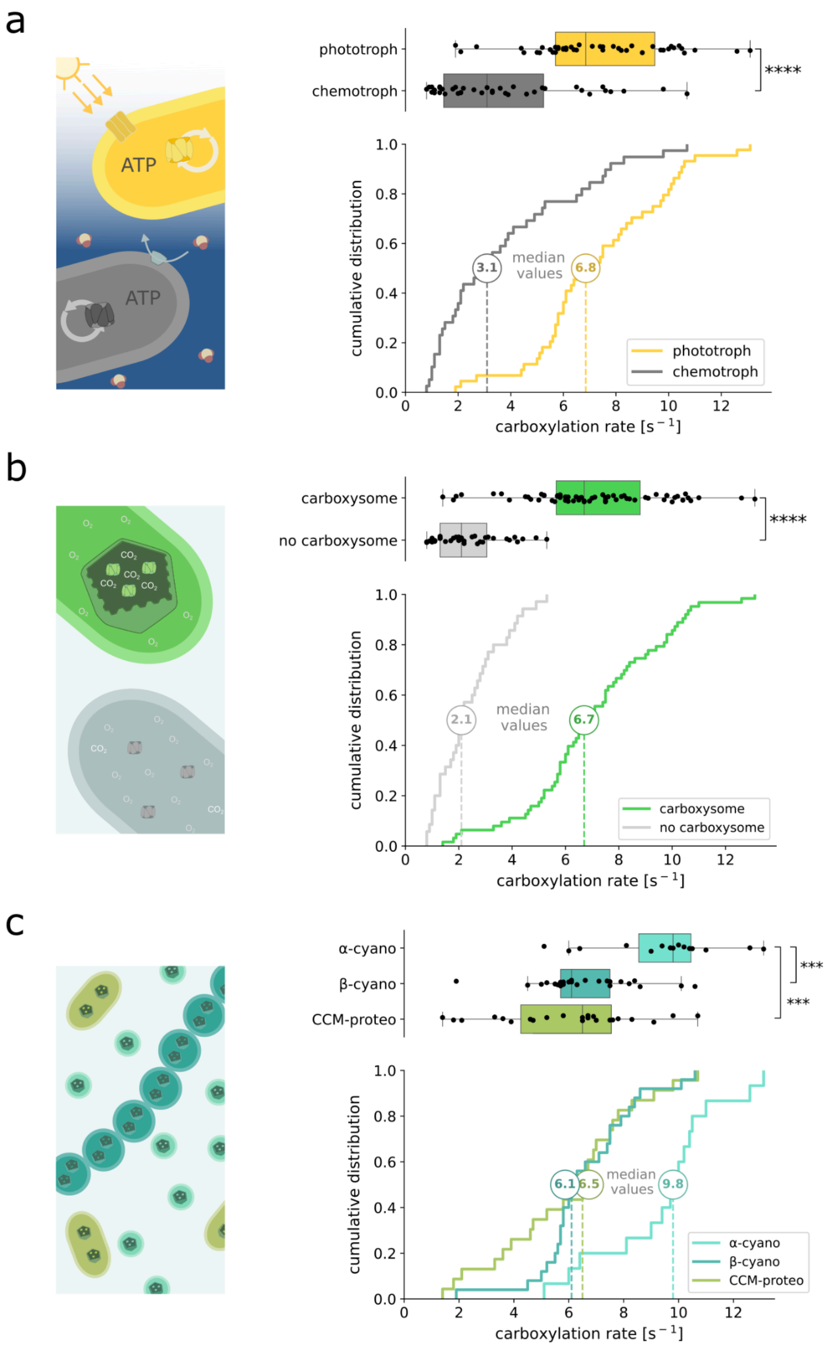

Appendix Figure S2. Comparative analysis of all measured rubisco rates from this study as a function of their associated biological parameters.

*(**A**–**C**) Box and cumulative distribution plots of all rubisco carboxylation rates from this study from different clusters: (**A**) chemo- and phototrophic bacterial rubiscos, (**B**) carboxysome-associated rubiscos and their counterparts, (**C**) α- and β- cyanobacterial, and carboxysome-associated proteobacterial rubiscos. Contrary to Fig. 2 where uniform sampling was ensured, present plots contain all rates measured in this study. Mann-Whitney U test (**A** and **B**) or Kruskal-Wallis followed by Dunn multiple comparison tests (**C**) were applied. ***$p < 0.001$, ****$p < 0.0001$. Legend abbreviations are as follows: α-cyano, α-cyanobacterial rubisco; β-cyano, β-cyanobacterial rubisco; CCM-proteo, carboxysome-associated proteobacterial rubisco.*

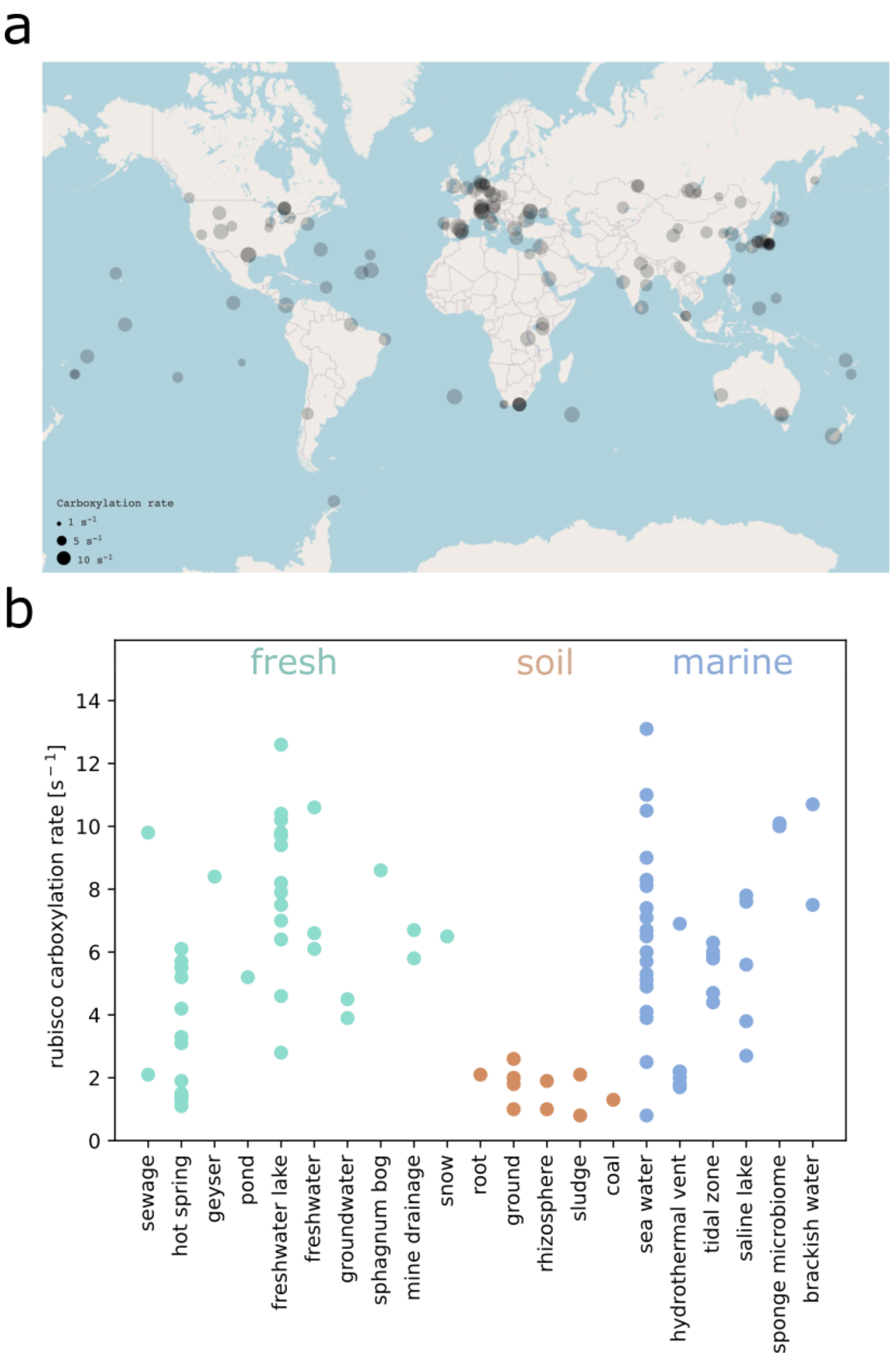

Appendix Figure S3. Environmental context of rubisco sequences tested in this study.
*(**A**) Map of rubisco carboxylation rates measured in this study, at the GPS coordinates of the biological samples associated with the cognate rubisco genes. Dots' area is proportional to measured carboxylation rates in vitro. (**B**) Rubisco carboxylation rate in function of its bacteria habitat. Environmental information of the biological sample containing each rubisco's sequence was collected and sorted into main habitats.*

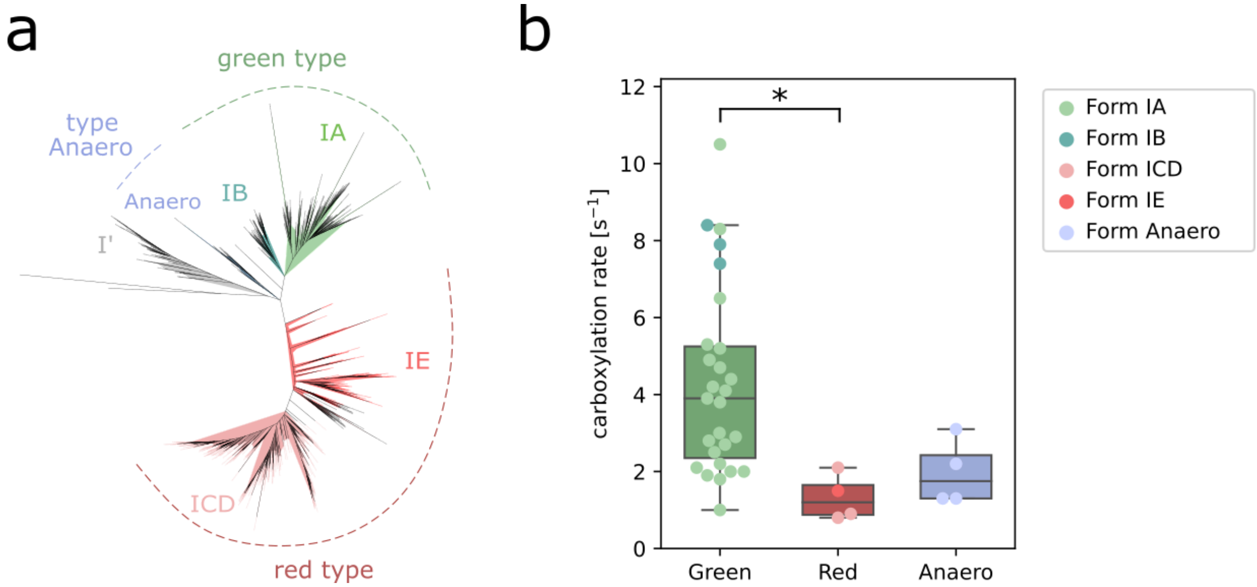

Appendix Figure S4. Green type rubiscos are faster than red type ones.
*(**A**) Form I rubisco large subunit phylogenetic tree showing the 3 distinct types: the "green", the "red", and the newly discovered "anaero" types (Schulz et al, 2022). (**B**) Box plot of carboxylation rates from rubiscos of the 3 types. To ensure unbiased study of each group, we selected a set of rubiscos uniformly covering their genetic diversity. Kruskal-Wallis followed by Dunn multiple comparison tests were applied. *$p < 0.05$.*

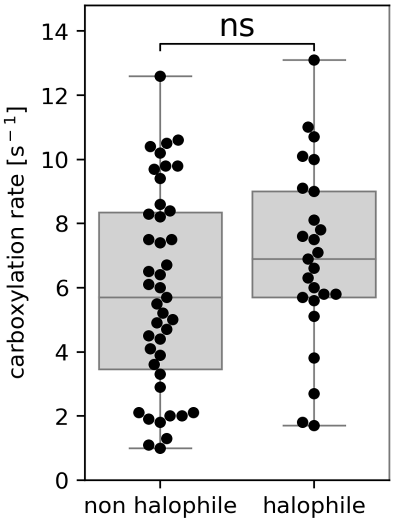

Appendix Figure S5. Rubisco carboxylation rate is not significantly associated with host halotolerance.
Bacteria relations to environment salinity were collected from literature and plotted against rubisco carboxylation rates measured in vitro. Mann-Whitney U test was applied. ns, non significant.

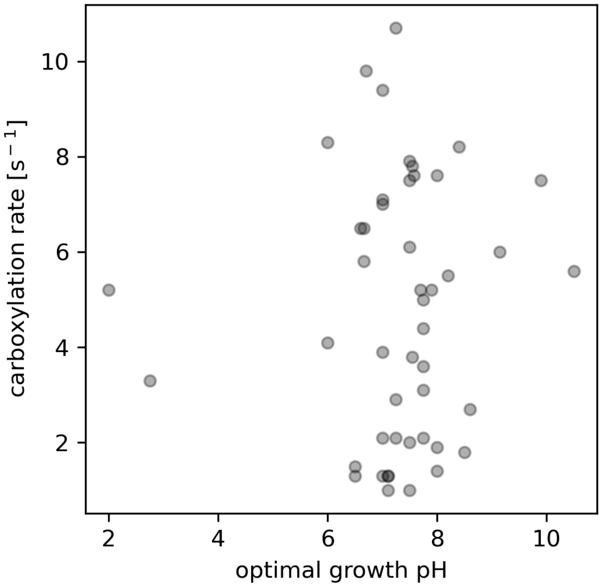

Appendix Figure S6. Rubisco carboxylation rate is not significantly associated with the optimal growth pH in the host environment.
Bacteria optimal pH were collected from literature and plotted against their rubisco carboxylation rate measured in vitro.

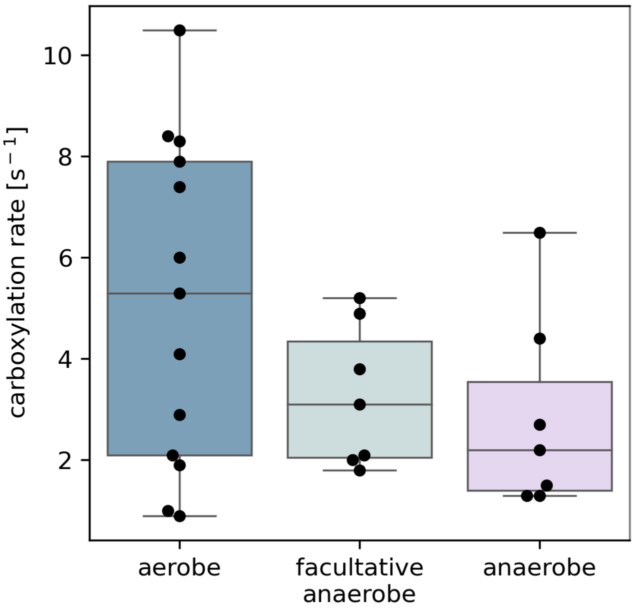

Appendix Figure S7. Rubisco carboxylation rate across bacteria with different oxygen tolerance.
Bacteria relations to oxygen were collected from literature and plotted against rubisco carboxylation rates measured in vitro. Kruskal-Wallis test indicated no significant variation between the groups.

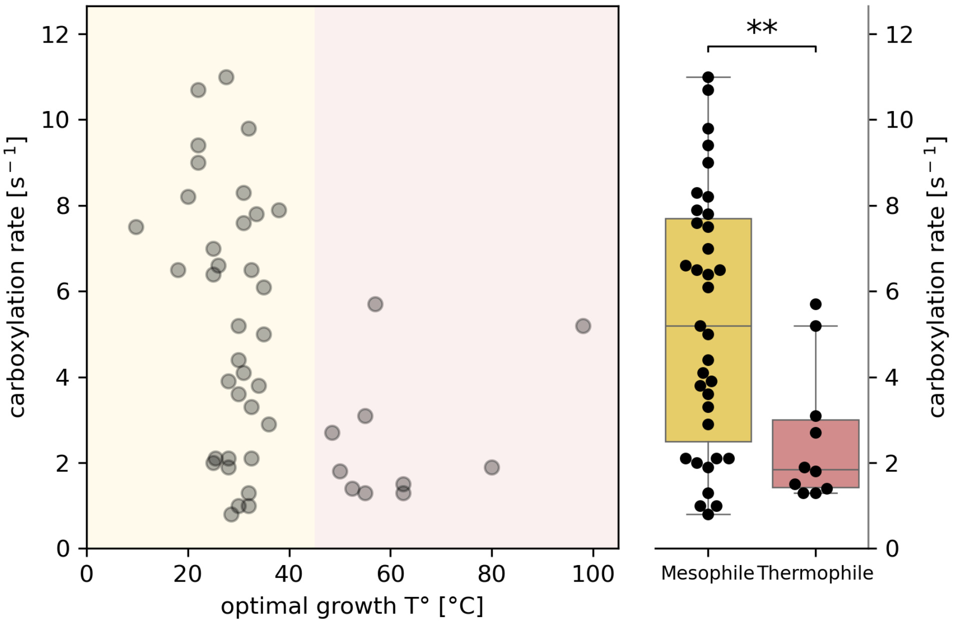

Appendix Figure S8. Rubisco carboxylation rate as a function of the optimal growth temperature of its host bacteria.

*Bacteria with optimal growth temperature below (yellow) and above (red) 45°C were considered as mesophiles and thermophiles respectively. Carboxylation rates of rubiscos from these 2 clusters were compared (right). Mann-Whitney U test was applied. **p < 0.01.*

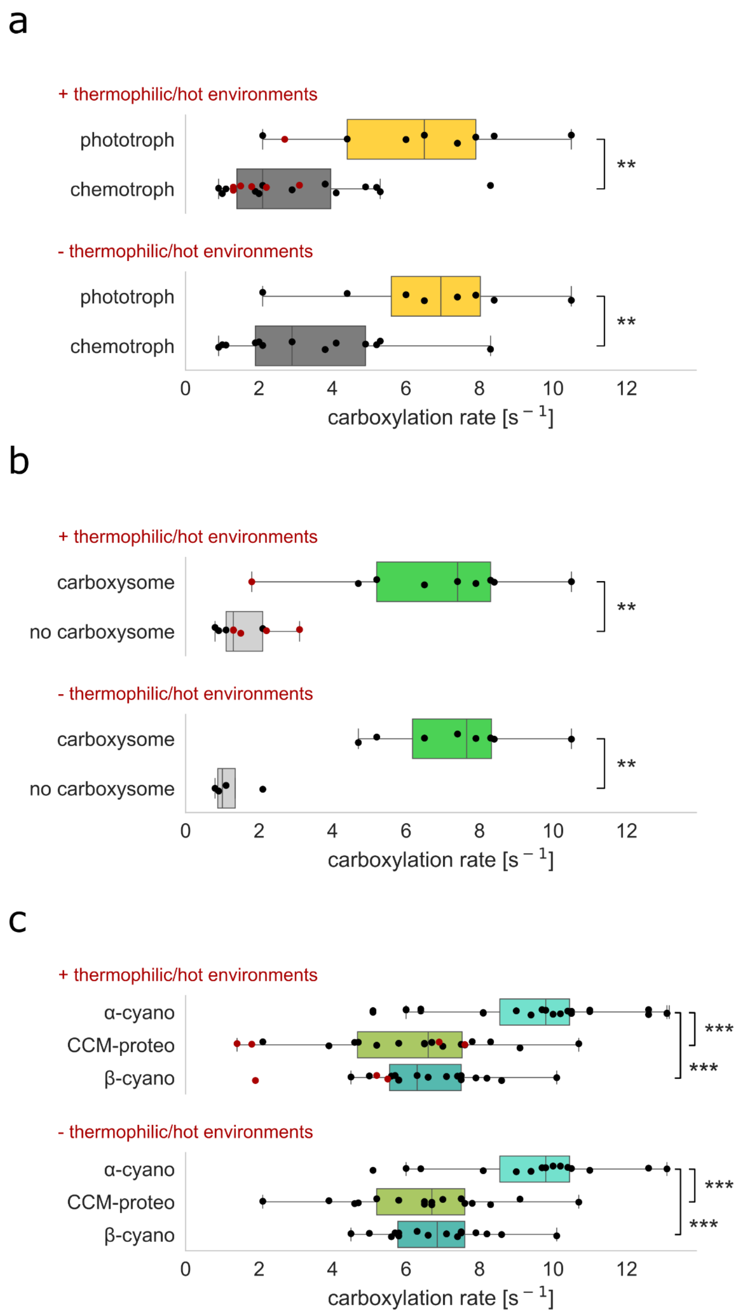

Appendix Figure S9. Comparative analysis of the biological parameters associated with fast carboxylating rubiscos with and without thermophilic or hot-environments-associated rubiscos. *(**A**–**C**) Box plots of rubisco carboxylation rates from different clusters with (upper parts) or without (lower parts) considering thermophilic or hot-environments-associated rubiscos (red dots). The different studied clusters are: (**A**) chemo- and phototrophic bacterial rubiscos, (**B**) carboxysome-associated rubiscos and their counterparts, (**C**) α- and β- cyanobacterial, and carboxysome-associated proteobacterial rubiscos. To ensure unbiased study of every group, class-representative rubiscos of each cluster were selected (see Materials and Methods). Mann-Whitney U test (**A** and **B**) or Kruskal-Wallis followed by Dunn multiple comparison tests (**C**) were applied. **$p < 0.01$, ***$p < 0.001$. Legend abbreviations are as follows: α-cyano, α-cyanobacterial rubisco; β-cyano, β-cyanobacterial rubisco; CCM-proteo, carboxysome-associated proteobacterial rubisco.*

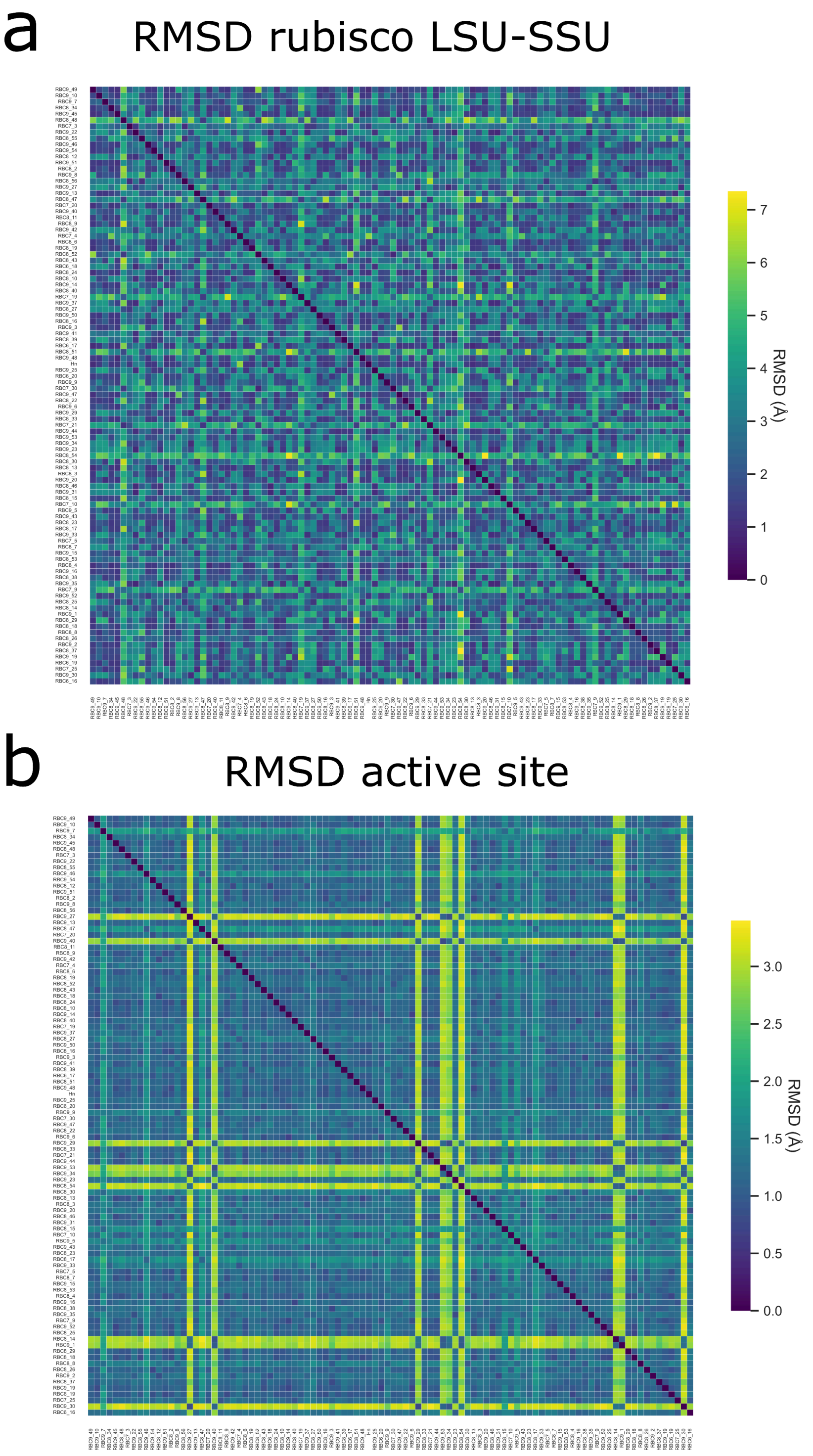

Appendix Figure S10. Comparative RMSD analysis of form I rubisco structures.
*(**A**–**B**) Color-coded heatmap representing the root-mean-square deviations of atomic positions (RMSD) values between rubisco structures (one large and small subunit - **A**) and between their active sites (19 active site amino acids - **B**). Each cell corresponds to the RMSD calculated between the rubisco pair annotated at the respective row and column by their ID (see Dataset EV2 for correspondence with protein accession number and species). The median RMSD value for all comparisons is 2.7 Å for the large and small subunit together, and 1.3 Å for the active site alone.*

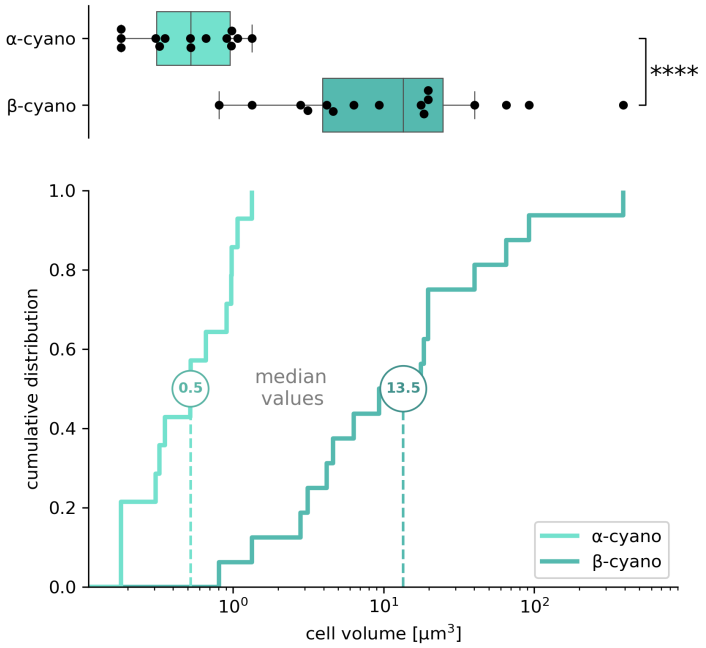

Appendix Figure S11. α-cyanobacteria are smaller than β-cyanobacteria.
*Box and cumulative distribution plots of the volume of α- and β- cyanobacterial cells. Size of cyanobacterial cells expressing rubiscos from Figure 2C were collected from the literature when possible and cell volume was calculated. Mann-Whitney U test was applied. ****$p < 0.0001$. Legend abbreviations are as follows: α-cyano, α-cyanobacteria; β-cyano, β-cyanobacteria.*

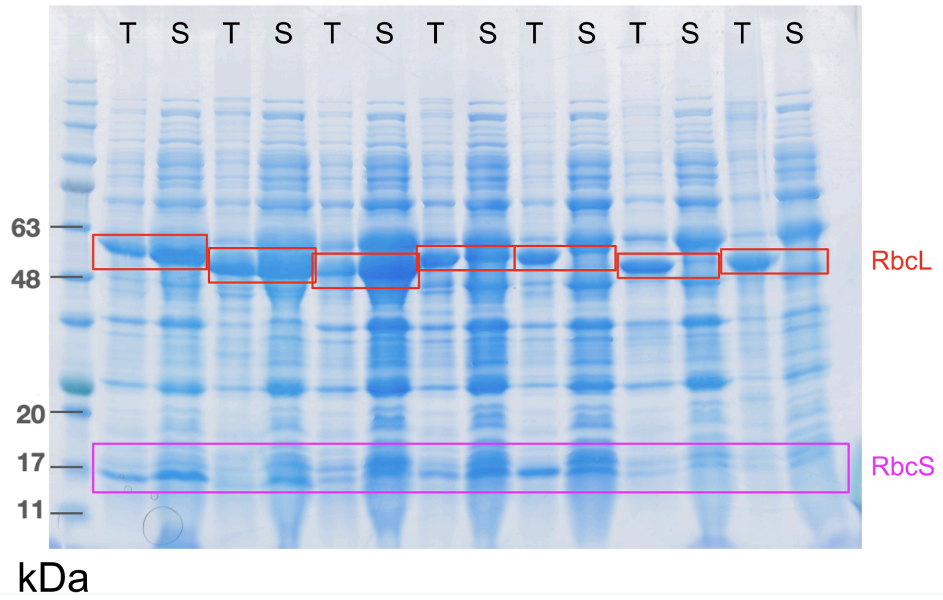

Appendix Figure S12. SDS-page analysis of form I rubisco variants expression and solubility.
A characteristic gel of total (T) and soluble (S) extracts from E. coli cells expressing 7 different form I rubisco variants. Rubisco large (RbcL - red boxes) and small (RbcS - purple box) are ≈50 and ≈15 kDa monomers respectively; protein ladder is BLUeye prestained (GeneDirex hy-labs).

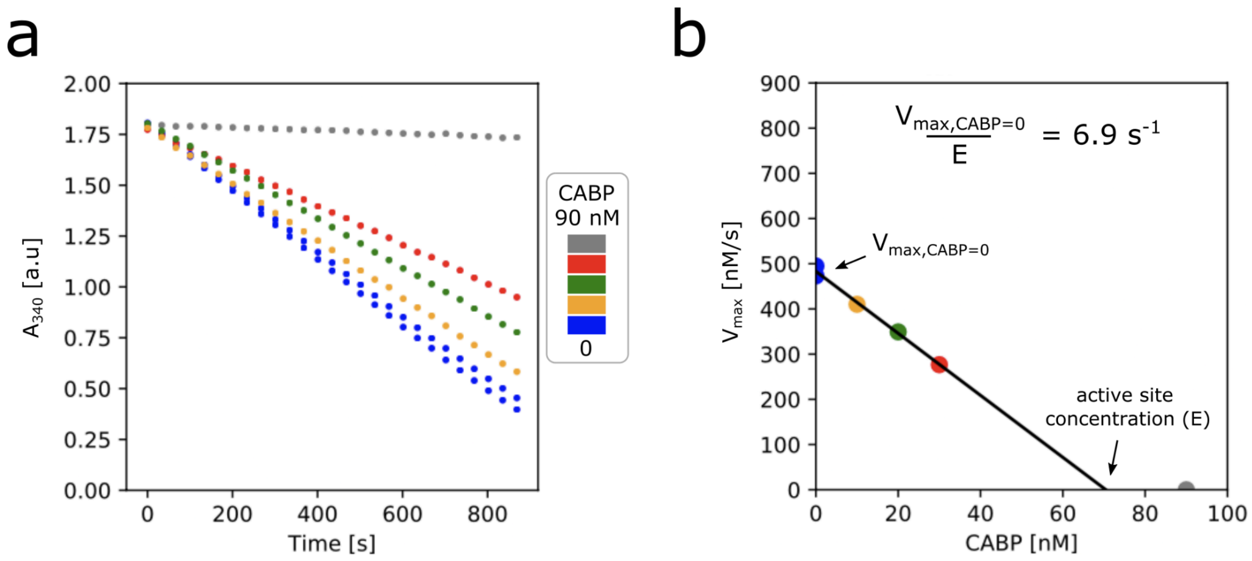

Appendix Figure S13. Spectroscopic enzymatic assay for measuring the carboxylation rate of rubisco from a cellular extract (as presented in Davidi et al, 2020).

*(**A**) Rubisco activity is coupled to NADH oxidation monitored at 340 nm. A gradient of CABP is used to gradually inhibit rubisco activity. Rubisco's maximal velocity (V_{max}) corresponds to half of the rate of NADH oxidation, which is the slope of the curves. (**B**) Rubisco's maximal velocity as a function of CABP concentration. The carboxylation rate of rubisco is given by dividing the maximal velocity without CABP (y-intercept) by the concentration of rubisco active sites (x-intercept). In this example, the carboxylation rate of R. rubrum (6.9 s^{-1}) is measured from a cellular extract without purification.*

Appendix Table S1. Recipe for the spectroscopic coupled assay.

Component	Assay concentration	Source
EPPS buffer pH 8.0	100 mM	Alfa Aesar (Cat # J61296)
MgCl2	20 mM	Sigma Aldrich (Cat # M2670-500G)
Dithiothreitol	0.5 mM	Bio Basic Canada inc. (Cat # DB0058)
ATP	2 mM	Sigma Aldrich (Cat # A3377-5G)
Phosphocreatine	10 mM	Sigma Aldrich (Cat # 27920-5G)
NADH	1.7 mM	Merck (Cat # 481913-1GM)
Carbonic anhydrase	0.1 mg/mL	Sigma Aldrich (Cat # C3934-100MG)
Creatine phosphokinase	20 U/mL	Sigma Aldrich (Cat # C3755-35KU)
Glyceraldehyde 3-phosphate dehydrogenase	20 U/mL	Sigma Aldrich (Cat # G2267-10KU)
3-Phosphoglyceric phosphokinase	20 U/mL	Sigma Aldrich (Cat # P7634-5KU)

Appendix Note S1: Rubisco expression and carboxylation assay from cell lysate

All 144 rubisco variants were cloned into a pET-29b vector and transformed into *E. coli* BL21 cells priorly transformed with a GroEL-GroES plasmid. Cells were grown to mid-log (OD_{600} ≈ 0.6). After induction of GroEL-GroES and rubisco expression, cells were incubated overnight at 23°C. Upon cell harvest, the expression and solubility of each rubisco variant was assessed by SDS-page of cell extracts before (total lysate) and after (soluble extract) centrifugation (Appendix Fig. S12).

Soluble extracts were then tested for carboxylation with a spectroscopic enzymatic assay. The assay was performed as described in Davidi *et al*, 2020. Namely, all assay components (see Appendix Table S1) were mixed and distributed into a 96-multiwell plate, except for the soluble cell extract and rubisco's substrate ribulose 1,5-bisphosphate (RuBP). After activation at 4% CO_2 and 0.4% O_2 for 15 minutes, soluble cell extracts were added to the wells and incubated for a further 15 minutes at the same gas conditions and at 30°C. The assay started upon the addition of RuBP to the wells and was immediately monitored for NADH oxidation (A340) in the gas-controlled plate reader. To measure the active site concentration, each variant was tested with 6 different concentrations of 2-C-carboxyarabinitol 1,5-bisphosphate (CABP) (0, 0, 10, 20, 30, and 90 nM) in parallel. CABP is a transition state analog and a stoichiometric rubisco inhibitor commonly used for active-site quantification (Kubien *et al*, 2011). Rubisco's carboxylation rate is determined by measuring the slope of the linear regression fitted between the reaction rates and the CABP concentrations (Appendix Fig. S13). We note that any NADH dehydrogenation due to other native *E. coli* proteins, while low (see the [CABP] = 90 nM gray curve of Appendix Fig. S13A), is not influencing the measurement of the carboxylation rate which relies on the differential V_{max} values at changing CABP concentrations (which should not affect the rates of these dehydrogenases).

Because it was not possible to estimate *a priori* the concentration of rubisco's active site in each extract, a first assay was performed with 10 µL of undiluted extract, often leading to saturation of this first assay. Following this, the concentration of each cell extract was adjusted through dilution to achieve a rubisco concentration that allowed measurable inhibition by CABP.

This pipeline enabled the determination of the carboxylation rate for ≈100 form I rubisco variants in a high-throughput manner.

Appendix Note S2: Effect of cell size on carbon uptake

Here we discuss the potential effect of cell size on carbon uptake by cyanobacteria. Existing literature suggests that cyanobacteria size is too small to have an effect on carbon uptake to the carboxysome (Mangan & Brenner, 2014; Mangan *et al*, 2016). However, these studies usually assume micron-sized cells, representing picocyanobacteria but not macrocyanobacteria (Sánchez-Baracaldo, 2015). We reevaluate this logic with a back of the envelope calculation with variable cell size (while not including other effects such as the differences in the efficiency of inorganic carbon pumps etc.).

We consider a spherical cell with a radius R and a concentration of inorganic carbon (C_i) in the medium $[C_i]_{ext}$.

Using Fick's first law, we can determine the amount of C_i taken by this cell per second. The equation is given by:

$\frac{dC_i}{dt} = -D_{C_i} \times S_r \times \frac{d[C_i]_r}{dr}$ (with $[C_i]_r$ the C_i concentration at a distance, r, away from the center of the cell. And S_r the surface of this r-radius sphere).

After differentiation, and with $[C_i]_r = [C_i]_{ext}(1 - \frac{R}{r})$ (considering C_i null at r = R)

$$\frac{dC_i}{dt} = -D_{C_i} \times 4\pi r^2 \times (-\frac{R \times [C_i]_{ext}}{r^2})$$
$$\frac{dC_i}{dt} = D_{C_i} 4\pi R\ [C_i]_{ext}$$

The values used are as follows (McNeil & Matsumoto, 2019; Falkowski & Raven, 2007):

- $D_{C_i} = D_{HCO_3^-} = 10^{-9}\ m^2.s^{-1}$ (among all inorganic carbon forms, we consider HCO_3^-, the most abundant form, and the one going into carboxysomes)
- $[C_i]\ in\ the\ ocean \approx 10^{-3}M = 1\ mol.m^{-3}$
- $[C_i]\ in\ a\ freshwater\ lake \approx 10^{-4}M = 10^{-1}\ mol.m^{-3}$
- $R = 10^{-6}\ m$ (assuming a cell radius in the order of a micron)

So $\frac{dC_i}{dt} \approx 10^{-16}\ mol.s^{-1}\ (or\ 10^{10}\ C_i.s^{-1})$ in the ocean.

And $\frac{dC_i}{dt} \approx 10^{-15}\ mol.s^{-1}\ (or\ 10^{9}\ C_i.s^{-1})$ in a freshwater lake.

Theoretically, the minimum time to get all the carbon to divide is $t_{min} = \frac{n_{C\ in\ a\ cell}}{\frac{d[C_i]}{dt}}$

In order to make it more realistic, one should consider 1. the inherent limitations of diffusion from the medium to the carboxysome (as not all cell surfaces are equipped with carbon transporters and aspects such as HCO_3^- diffusion and pool renewal may not be optimized in nature) and 2.

the simultaneous loss of carbon atoms by cells through processes like respiration and fermentation, we propose a more realistic estimation by multiplying the minimum time by a factor of 10.

So, more probably, $t_{min} = 10 \times \frac{n_{C\ in\ a\ cell}}{\frac{d[C_i]}{dt}}$

For a cell with a radius of 1μm, the number of carbon atoms in the cell is $n_{C\ in\ a\ cell} \approx 10^{10}$.

So $t_{min} = 10\ s$ for a cell with a radius of 1μm that thrives in the ocean and $100\ s$ in a freshwater lake.

Cyanobacteria have doubling times in the order of once a day. So, in both cases, $t_{min} << T_d$ for a cell with a radius of 1 μm.

But some cyanobacteria (like *Limnoraphis robusta CS-951*) have a radius of 10 μm*.

For these cells, $n_{C\ in\ a\ cell} \approx 10^{13}$ and $\frac{dC_i}{dt} \approx 10^{11}\ C_i.s^{-1}$ in the ocean and $10^{10}\ C_i.s^{-1}$ in a freshwater lake.

So, for such cells, $t_{min} = 10^3\ s \approx 0.3\ h$ in the ocean and $3\ h$ in a freshwater lake.

We can therefore see that, in this last situation, the minimum time to get all the carbon to divide approaches the cell doubling time. This may be accentuated in cases where dissolved inorganic carbon concentrations are lower than typically measured for photic zones in oceanic or freshwater environments. In the case of α-cyanobacteria which were shown to dominate their beta counterparts not only in the oceans, but also in freshwater environments (Cabello-Yeves *et al*, 2022), their small size could therefore contribute to better supplying CO_2 to the rubisco. Finally, this smaller size (and increased cell surface-to-volume ratio) may potentially enhance the acquisition of other resources, including light. This, in turn, could further contribute to fueling the energy-consuming CCM and better concentrate CO_2 around rubisco.

*Actually, these cells are probably even worse carbon uptakers as they are stacked cylinders with a radius of 10 μm (and with a trichome's length going up to 5 mm)

References

Cabello-Yeves PJ, Scanlan DJ, Callieri C, Picazo A, Schallenberg L, Huber P, Roda-Garcia JJ, Bartosiewicz M, Belykh OI, Tikhonova IV, *et al* (2022) α-cyanobacteria possessing form IA RuBisCO globally dominate aquatic habitats. *ISME J*

Davidi D, Shamshoum M, Guo Z, Bar-On YM, Prywes N, Oz A, Jablonska J, Flamholz A, Wernick DG, Antonovsky N, *et al* (2020) Highly active rubiscos discovered by systematic interrogation of natural sequence diversity. *EMBO J* 39: e104081

Falkowski PG & Raven JA (2007) Aquatic Photosynthesis: Second Edition STU - Student edition. Princeton University Press

Kubien DS, Brown CM & Kane HJ (2011) Quantifying the amount and activity of Rubisco in leaves. *Methods Mol Biol* 684: 349–362

Mangan NM & Brenner MP (2014) Systems analysis of the CO2 concentrating mechanism in cyanobacteria. *Elife* 3: e02043

Mangan NM, Flamholz A, Hood RD, Milo R & Savage DF (2016) pH determines the energetic efficiency of the cyanobacterial CO_2 concentrating mechanism. *Proceedings of the National Academy of Sciences* 113: E5354–E5362

McNeil BI & Matsumoto K (2019) 1 - The changing ocean and freshwater CO2 system. In *Fish Physiology*, Grosell M Munday PL Farrell AP & Brauner CJ (eds) pp 1–32. Academic Press

Sánchez-Baracaldo P (2015) Origin of marine planktonic cyanobacteria. *Sci Rep* 5: 1–10

Schulz L, Guo Z, Zarzycki J, Steinchen W, Schuller JM, Heimerl T, Prinz S, Mueller-Cajar O, Erb TJ & Hochberg GKA (2022) Evolution of increased complexity and specificity at the dawn of form I Rubiscos. *Science* 378: 155–160
