# Supplementary material for: A systematic exploration of bacterial form I rubisco maximal carboxylation rates
Source: EMBO J. 2024 May 28;43(14):13. doi: 10.1038/s44318-024-00119-z (PMC11251275; doi:10.1038/s44318-024-00119-z)
Supplement: Supplementary file 8 — Expanded View Figures [file 44318_2024_119_MOESM8_ESM.pdf]

Expanded View Figures

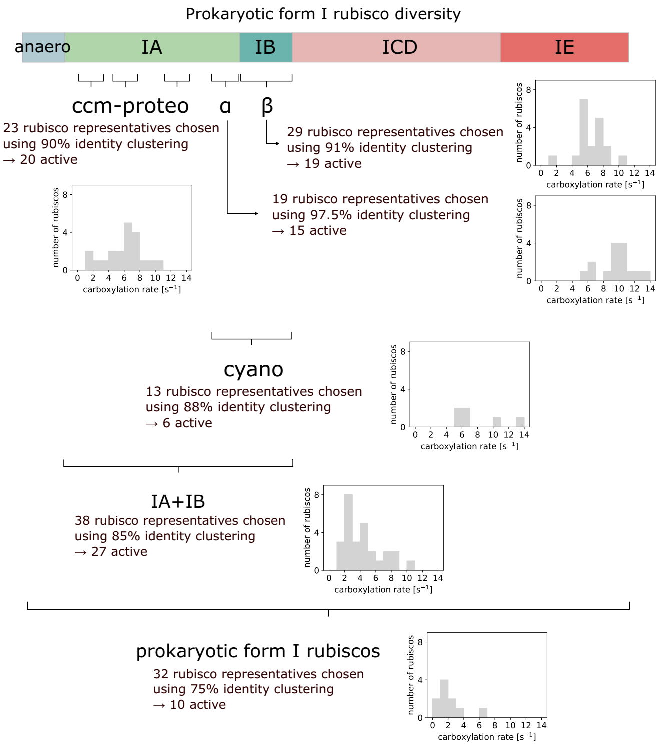

Figure EV1. Sequential screening strategy used for the selection of rubisco variants for characterization.

An iterative clustering approach was employed to screen the totality and specific subgroups of the form I rubisco family. The number of active rubiscos and measured carboxylation rates are indicated at each step.

 © The Author(s)

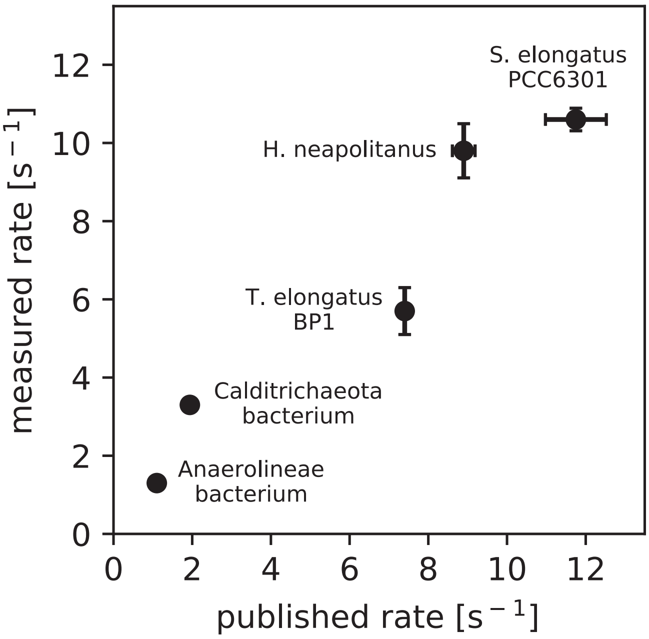

Figure EV2. Carboxylation rates measured in this study have similar ranking as rates published in the literature in spite of different techniques and conditions.

Values in both axes are not supposed to be equal as those measured in this study result from coupled assays, which tend to underestimate the rates compared to direct assays used in the literature, and on the other side they were performed at 30 °C, resulting in faster rates compared to literature measurements done at 25 °C.

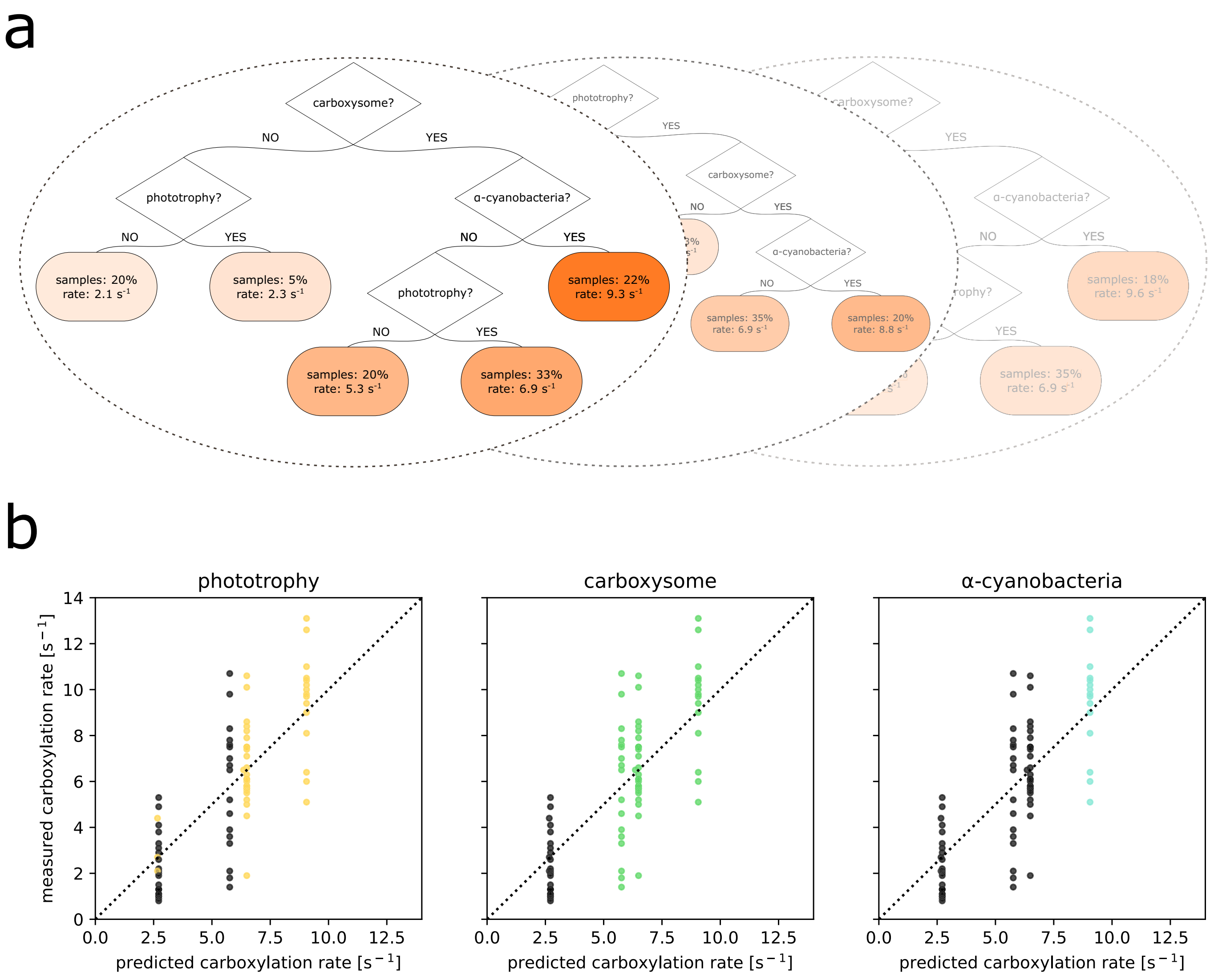

Figure EV3. Random forests modeling of form I rubisco carboxylation rate as a function of the main parameters from this study.

(**A**) Examples of decision trees out of the hundred trained in the random forests. (**B**) Measured against predicted carboxylation rates for the three most influential features according to the model. Each dot is color-coded based on the value of the respective feature (yellow/black: phototrophic/chemotrophic; green/black: carboxysome/non-carboxysome associated; cyan/black: α-cyanobacterial/non-α-cyanobacterial rubisco). RMSE = 2.1 s^{-1}; average explained variance score = 0.55.

© The Author(s)
